# Supplementary material for: Microbial trend analysis for common dynamic trend, group comparison, and classification in longitudinal microbiome study
Source: BMC Genomics. 2021 Sep 15;22:667. doi: 10.1186/s12864-021-07948-w (PMC8442444; doi:10.1186/s12864-021-07948-w)
Supplement: Supplementary file 1 — Additional file 1Section S1: Simulation study based on the simulation design of the perturbation method. Section S2: Comparison between relative abundance and log-ratio transformed relative abundances. Section S3: Principal trend analysis. Section S4: Laplacian matrix construction. Section S5: The derivation of Algorithm 1. Section S6: Comparisons among the proposed MTA, Permuspliner, MetaLonDA, and LonGP. Section S7: Sparsity evaluation of MTA. [file 12864_2021_7948_MOESM1_ESM.pdf]

Additional file 1: Additional file 1 has 8 sections as below.

Section S1: Simulation study based on the simulation design of the perturbation method

Without loss of generality, we assume that there is only one different trend between control and case groups and 3 dominant taxa that contribute to it. As described in the "Simulation design" section, we denote the set of their indices as $\wedge_1$ and their corresponding deviations as $\boldsymbol{\beta}^1_{.t} = (\beta^1_{1t}, \beta^1_{2t}, \beta^1_{3t})'$. Then $\boldsymbol{\beta}^1_{.t}$ is assigned as the following perturbations based on the perturbing values in Shields-Cutler *et al.* (2018), instead of equation (2).

$$\boldsymbol{\beta}^1_{.t} = e \times (-2, 4, -2)' \times \boldsymbol{z}[t] \times \min(1, \beta_{0p}/2),\ p \in \wedge_1,$$

where e is the magnitude of perturbation and was assigned to 1 and 2 respectively. $\boldsymbol{z}$ is the vector of the perturbing values. Here we consider two sets of values for $\boldsymbol{z}$: $(0, 0.3, 0.45, 0.2, -0.3, 0.3, -0.3, -0.2, -0.1, 0)'$ or $(0, 0.1, 0.2, 0.3, 0.4, 0.3, 0.2, 0.1, 0.1, 0)'$ for $T = 10$, and $(0, 0.2, 0.5, 0.3, 0.2, -0.2, -0.4, -0.4, -0.2, 0.2, 0.5, 0.2, -0.3, -0.4, -0.2, 0.2, 0.4, 0.2, 0.1, 0)'$ or $(0, 0.05, 0.1, 0.2, 0.2, 0.2, 0.3, 0.3, 0.2, 0.2, 0.2, 0.2, 0.3, 0.3, 0.2, 0.2, 0.2, 0.2, 0.1, 0)'$ for $T = 20$. The first set assumes that the causal taxa have different changing directions while the second set assumes that the causal taxa have the same changing direction at all the time points. In addition, the number of cases and controls N and the number of resamplings R in Algorithm 2 are the same as those in the "Simulation study" section.

Section S2: Comparison between relative abundance and log-ratio transformed relative abundances

The compositional nature of microbiome data has raised many debates in the field. The constant sum constraint can lead traditional statistical methods to produce spurious correlations and errant results. Log-ratio normalizations have been commonly used to address microbial compositionality and have achieved considerable progress. But the problem caused by the compositionality is still open in the longitudinal microbiome study. Here we constructed some simulation studies to illustrate the performance of the proposed MTA framework using relative abundance, and three transformed relative abundances with log, additive log-ratio (ALR), and centered log-ratio (CLR) transformations, respectively. Specifically, we consider scenarios 1-3 with sample size $N = 20$ and the number of time points $T = 10$ under two simulation designs: the first is the same as the one we used in the "Simulation design" section; the second one is constructed to be in favor of the transformed methods and based on the Dirichlet distribution with a different modeling. The mean relative abundance of the pth taxon for a subject at the tth time point is assigned as below, different from equation (1) in the first design,

$$E[O_{pt}] = \frac{\gamma_{pt}}{\sum_{p=1}^{P} \gamma_{pt}}, \;\log(\gamma_{pt}) \;=\; \beta^l_{0p} + \beta^l_{pt},\; p = 1, \ldots, P,\; t = 1, \ldots, T,$$

where $\boldsymbol{\beta}^l_0 = (\beta^l_{01}, \ldots, \beta^l_{0P})'$ represents the log-transformed baseline relative abundances for $P = 35$ taxa and they were set as the corresponding estimates from the control mice using R package dirmult (Tvedebrink, 2009). β^l_{pt} is the deviation of the pth taxon from the log-transformed baseline relative abundance β^l_{0p} at the tth time point, which contributes to the difference between control and case groups. The deviations of 5 dominant taxa are assigned by equations (S1) and (S2) respectively,

$$\boldsymbol{\beta}^{1l}_{.t} = (-1, 2, -1)' \times \frac{1}{2}\sin(t); \tag{S1}$$

$$\boldsymbol{\beta}^{2l}_{.t} = (-1, 1)' \times \frac{1}{2}\sin(2t). \tag{S2}$$

Furthermore, the remaining parameters are set as the same as those in the "Simulation design" section.

Figure S13 presents the estimated powers of the proposed MTA method using the relative abundances, the log, ALR, and CLR transformations under two simulation designs respectively. We observe that the power using the relative abundances directly is highest under any scenario and using relative abundance makes the proposed MTA method capture more dynamic information and elucidate more accurate difference between control and case groups. The reason that MTA based on the relative abundance has better performance is twofold. First, MTA evaluates the dynamic difference between cases and controls by comparing the trends extracted from a difference array $\mathbf{G}^*$ to a noise trend which is randomly arranged around 0 across all time points, rather than directly compare the dynamic trends extracted from cases and controls, respectively. Second, MTA employs matrix factorization and regularization techniques to analyze all taxa together and identify the dominant taxa simultaneously, by assuming that the microbiota act as an integrated microbial community. This strategy releases the correlations among taxa due to compositionality to some extent (Zou *et al.*, 2006; Maxwell *et al.*, 2019). We recognize that all results above are based on the simulation studies, rather than on theoretical consideration. We also include log, ALR, and CLR transformations in the MTA R package as options and let the users decide the transformation procedure based on their studies.

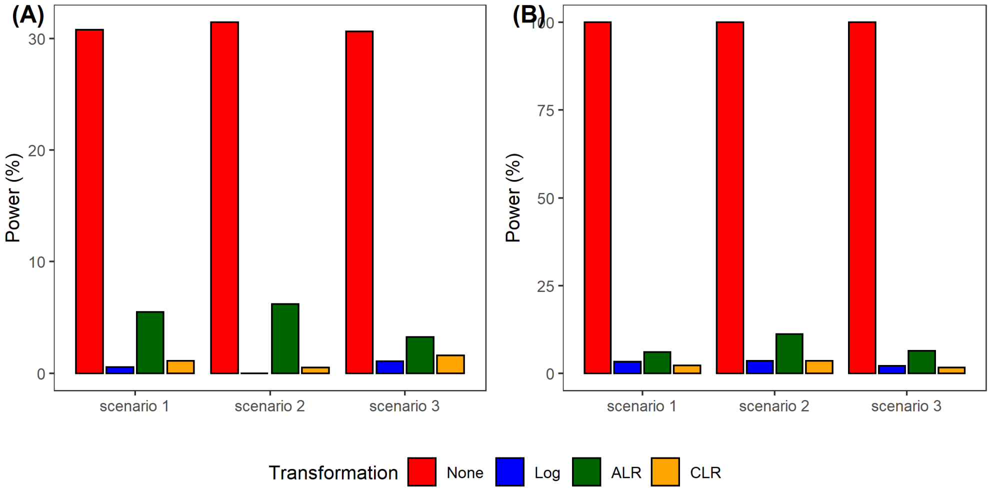

FIG S13 Estimated powers (%) of the proposed MTA method for testing the microbial trend difference between case and control groups using different transformed relative abundances, with sample size $N = 20$ and the number of time points $T = 10$. Specifically, the proposed MTA method was applied on the relative abundances directly (None; red bar), the log transformation (Log; blue bar), ALR (additive log-ratio transformation; darkgreen bar), and CLR (centered log-ratio transformation; orange bar), respectively under the scenarios 1-3. (A) Scenarios 1-3 were constructed with the simulation design described in the “Simulation design” section. (B) Scenarios 1-3 were constructed with the simulation design described in Additional file 1, Section S2.

Section S3: Principal trend analysis

Zhang and Davis (2013) proposed the PTA method for analyzing the time-course gene expression data. Being an unsupervised approach, PTA aims to extract the group-level principal trends of time-course gene expression data from a group of subjects and identify genes that make dominant contributions to the principal trends. They suppose there are N subjects in a given group. Let Y_{npt} be the gene expression of the pth gene of subject n at time point t, $p = 1, \dots, P$, and $t = 1, \dots, T$, and $\boldsymbol{Y}_n$ denote a $P \times T$ matrix of observations of subject n; $\boldsymbol{F} = (\boldsymbol{f}_1, \dots, \boldsymbol{f}_M)$ be a $P \times M$ matrix of factor scores; $\boldsymbol{C} = (\boldsymbol{c}_1, \dots, \boldsymbol{c}_M)'$ be an $M \times (T+2)$ matrix of spline coefficients; $\boldsymbol{B} = \{B_i(t)\}_{t,i=1}^{t=T,i=T+2}$ be a $T \times (T+2)$ matrix containing the cubic spline basis; $\boldsymbol{Q} = \boldsymbol{C}\boldsymbol{B}'$ denote a $M \times T$ matrix presenting the top M common trends across T time points. The underlying model is expressed as

$$\boldsymbol{Y}_n = \boldsymbol{F}\boldsymbol{C}\boldsymbol{B}' + \boldsymbol{E}_n, \ n = 1, \dots, N$$

where, $\boldsymbol{E}_n$ denotes the error term for subject n. Zhang and Davis (2013) estimated the parameters $\boldsymbol{F}$ and $\boldsymbol{C}$ by solving the optimization problem below

$$\min_{\boldsymbol{F},\boldsymbol{C}} L(\boldsymbol{F}, \boldsymbol{C}|\boldsymbol{Y}_n) = \sum_{n=1}^{N} \|\boldsymbol{Y}_n - \boldsymbol{F}\boldsymbol{C}\boldsymbol{B}'\|_F^2, \tag{S3}$$

$$\text{subject to } \ \boldsymbol{C}\boldsymbol{\Omega}\boldsymbol{C}' \leq a_1, \|\boldsymbol{F}\|_1 \leq a_2, \ \text{and } \|\boldsymbol{F}\|_2^2 = 1,$$

where $\|\cdot\|_F$ is the Frobenius norm, $\boldsymbol{\Omega} = \{\Omega_{ij}\}_{i,j=1}^{i,j=T+2}$ is a $(T+2) \times (T+2)$ matrix with $\Omega_{ij} = \int B_i''(t) B_j''(t) dt$, $i, j = 1, \dots, T+2$. a_1 and a_2 control the smoothness of trends and sparsity on genes, respectively. Using Langrange multipliers, the optimization problem (S3) is rewritten as

$$\min_{\boldsymbol{F},\boldsymbol{C}} \left[L(\boldsymbol{F}, \boldsymbol{C}|\boldsymbol{Y}_n) + \lambda_1 \boldsymbol{C}\boldsymbol{\Omega}\boldsymbol{C}' + \lambda_2 \|\boldsymbol{F}\|_1 \right], \tag{S4}$$

subject to $\|\boldsymbol{F}\|_2^2 = 1$, where $\lambda_1 (\geq 0)$ and $\lambda_2 (\geq 0)$ are tuning parameters that control

smoothness of the common trends and sparsity on genes, respectively. Zhang and Davis (2013) demonstrated the optimization problem (S4) is not convex, but biconvex and proposed an iterative algorithm (Algorithm S1) to obtain the parameter estimates $\hat{\boldsymbol{F}}$ and $\hat{\boldsymbol{C}}$.

Algorithm S1 Nonlinear iterative algorithm for PTA

Input: the observed data $\boldsymbol{Y}_n$ ($n = 1, \ldots, N$), the number of common trends M, and the fixed tuning parameters λ_1 and λ_2

For $m = 1, 2, \ldots, M$

Initialize: $\boldsymbol{Y}_n^1 = \boldsymbol{Y}_n, n = 1, \ldots, N$

For $i = 1, 2, \ldots$

Initialize: $\hat{\boldsymbol{c}}_m^{(1)} = \boldsymbol{0}$, and $\hat{\boldsymbol{f}}_m^{(1)}$ with $\|\hat{\boldsymbol{f}}_m^{(1)}\|_2 = 1$ as the first estimated factor score vector from the mean matrix $\frac{1}{N}\sum_{n=1}^{N} \boldsymbol{Y}_n^m$.

Step 1: $\hat{\boldsymbol{c}}_m^{(i+1)} = (N\boldsymbol{B}'\boldsymbol{B} + \lambda_1\boldsymbol{\Omega})^{-1}\boldsymbol{B}'\left(\sum_{n=1}^{N}\boldsymbol{Y}_n^m\right)'\hat{\boldsymbol{f}}_m^{(i)}$

Step 2: $\hat{\boldsymbol{f}}_m^{(i+1)} = \text{Soft}\left(\left(\sum_{n=1}^{N}\boldsymbol{Y}_n^m\right)\boldsymbol{B}\hat{\boldsymbol{c}}_m^{(i+1)}, \frac{\lambda_2}{2}\right)$, where $\text{Soft}(a, b) = \text{sign}(a)(|a| - b)_+$ is the soft-thresholding operator.

Step 3: $\hat{\boldsymbol{f}}_m^{(i+1)} = \hat{\boldsymbol{f}}_m^{(i+1)}/\|\hat{\boldsymbol{f}}_m^{(i+1)}\|_2$

If both $\hat{\boldsymbol{f}}_m$ and $\hat{\boldsymbol{c}}_m$ are convergent, **then** break; $\hat{\boldsymbol{Q}}_m = \boldsymbol{B}\hat{\boldsymbol{c}}_m$ is the estimated m^{th} common trend and $\hat{\boldsymbol{f}}_m$ is the corresponding estimated factor scores.

$\boldsymbol{Y}_n^{m+1} = \boldsymbol{Y}_n^m - \hat{\boldsymbol{f}}_m\hat{\boldsymbol{c}}_m\boldsymbol{B}'$

With the estimates $\hat{\boldsymbol{F}}$ and $\hat{\boldsymbol{C}}$, PTA identifies the group-level time-course patterns $\hat{\boldsymbol{Q}} = \hat{\boldsymbol{C}}\boldsymbol{B}'$ shared by all subjects, and the dominant genes contributing to the common patterns which have non-zero estimated factor scores $\hat{\boldsymbol{F}}$. Besides, cross-validation is used to select the tuning parameters in the algorithm of PTA.

Section S4: Laplacian matrix construction

Given phylogenetic tree, we can calculate a matrix of patristic distances for all pairs of taxa. The patristic distance between any two taxa is defined as the length sum of all the branches that connect these two taxa and it measures the evolutionary divergence. Then the adjacency matrix $\boldsymbol{A}$ is based on the normalized patristic distance $\boldsymbol{D} = (d_{jk})$ as the following:

$$a_{jk} = \begin{cases} 1 & j = k \\ \phi(d_{jk}) & j \neq k \end{cases},$$

where $\phi(x)$ is a decreasing function and is chosen as $\phi(x) = 1-x$ in this paper. Larger a_{jk} represents OTU j and OTU k have closer evolutionary relationship. Finally Laplacian matrix $\boldsymbol{L}$ is defined as

$$\boldsymbol{L} = \boldsymbol{D}_d - \boldsymbol{A},$$

where $\boldsymbol{D}_d = \text{diag}(d_1, \dots, d_P)$ and $d_j = \sum_{k=1}^{P} a_{jk}$. The Laplacian penalty weights the OTUs closely linked on the phylogenetic tree to have similar effects on the extracted common trends.

Section S5: The derivation of Algorithm 1

Following Zhang and Davis (2013), the sum of squared errors $L(\boldsymbol{F}, \boldsymbol{C}|\boldsymbol{Y}_n)$ is illustrated as

$$L(\boldsymbol{F}, \boldsymbol{C}|\boldsymbol{Y}_n) = \sum_{n=1}^{N} \mathrm{tr}(\boldsymbol{Y}_n\boldsymbol{Y}_n') - 2\sum_{k=1}^{K} \boldsymbol{c}_k'\boldsymbol{B}' \left(\sum_{n=1}^{N} \boldsymbol{Y}_n'\right) \boldsymbol{f}_k + N\sum_{k=1}^{K} \boldsymbol{f}_k' \left(\boldsymbol{c}_k'\boldsymbol{B}'\boldsymbol{B}\boldsymbol{c}_k \bigotimes \boldsymbol{I}_P\right) \boldsymbol{f}_k.$$

So, in the case $K = 1$, $\boldsymbol{f}$ and $\boldsymbol{c}$ can be estimated by solving the following problem

$$\min\left\{-2\boldsymbol{c}'\boldsymbol{B}'\left(\sum_{n=1}^{N}\boldsymbol{Y}_n'\right)\boldsymbol{f} + N\boldsymbol{c}'\left(\boldsymbol{B}'\boldsymbol{B}\bigotimes\boldsymbol{f}'\boldsymbol{f}\right)\boldsymbol{c} + \lambda_1\boldsymbol{c}\boldsymbol{\Omega}\boldsymbol{c}' + \lambda_2\|\boldsymbol{f}\|_1 + \lambda_3\boldsymbol{f}'\boldsymbol{L}\boldsymbol{f}\right\} \tag{S5}$$

or the equivalent problem

$$\min\left\{-2\boldsymbol{c}'\boldsymbol{B}'\left(\sum_{n=1}^{N}\boldsymbol{Y}_n'\right)\boldsymbol{f} + N\boldsymbol{f}'\left(\boldsymbol{c}'\boldsymbol{B}'\boldsymbol{B}\boldsymbol{c}\bigotimes\boldsymbol{I}_P\right)\boldsymbol{f} + \lambda_1\boldsymbol{c}\boldsymbol{\Omega}\boldsymbol{c}' + \lambda_2\|\boldsymbol{f}\|_1 + \lambda_3\boldsymbol{f}'\boldsymbol{L}\boldsymbol{f}\right\} \tag{S6}$$

subject to $\|\boldsymbol{f}\|_2^2 = 1$.

With a fixed value $\hat{\boldsymbol{f}}$, we set the derivative of expression (S5) to 0, and solve for $\boldsymbol{c}$:

$$\boldsymbol{0} = -2\boldsymbol{B}'\left(\sum_{n=1}^{N}\boldsymbol{Y}_n'\right)\hat{\boldsymbol{f}} + 2N\left(\boldsymbol{B}'\boldsymbol{B}\bigotimes\hat{\boldsymbol{f}}'\hat{\boldsymbol{f}}\right)\boldsymbol{c} + \lambda_1\boldsymbol{\Omega}\boldsymbol{c}.$$

Then

$$\hat{\boldsymbol{c}} = \left(N\boldsymbol{B}'\boldsymbol{B} + \lambda_1\boldsymbol{\Omega}\right)^{-1}\boldsymbol{B}'\left(\sum_{n=1}^{N}\boldsymbol{Y}_n'\right)\hat{\boldsymbol{f}}.$$

With the estimated value $\hat{\boldsymbol{c}}$, we set the derivative of expression (S6) to 0, and solve for $\boldsymbol{f}$:

$$\boldsymbol{0} = -2\hat{\boldsymbol{c}}'\boldsymbol{B}'\left(\sum_{n=1}^{N}\boldsymbol{Y}_n'\right) + 2N\boldsymbol{f}'\left(\hat{\boldsymbol{c}}'\boldsymbol{B}'\boldsymbol{B}\hat{\boldsymbol{c}}\bigotimes\boldsymbol{I}_P\right) + \lambda_2\boldsymbol{\Gamma} + 2\lambda_3\boldsymbol{f}'\boldsymbol{L}$$

subject to $\|\boldsymbol{f}\|_2^2 = 1$, where $\boldsymbol{\Gamma} = [\gamma_1, \ldots, \gamma_P]$ and $\gamma_p = \begin{cases} \mathrm{sgn}(f_p) & f_p \neq 0 \\ \in [-1, 1] & \text{otherwise} \end{cases}$. Thus the

estimate $\hat{\boldsymbol{f}}$ is

$$
\begin{aligned}
\hat{\boldsymbol{f}}^0 &= \left[\mathrm{Soft}\left(\hat{\boldsymbol{c}}\boldsymbol{B}'\left(\sum_{n=1}^{N}\mathbf{Y}_n'\right), \frac{\lambda_2}{2}\right)(Nh\boldsymbol{I}_P + \lambda_3\boldsymbol{L})^{-1}\right]', \\
\hat{\boldsymbol{f}} &= \frac{\hat{\boldsymbol{f}}^0}{\|\hat{\boldsymbol{f}}^0\|_2},
\end{aligned}
$$

where $h = \text{scalar}\,(\hat{\boldsymbol{c}}'\boldsymbol{B}'\boldsymbol{B}\hat{\boldsymbol{c}})$, $\mathrm{Soft}(a, b) = \mathrm{sign}(a)(|a| - b)_+$ is the soft thresholding operator.

Section S6: Comparisons among the proposed MTA, Permuspliner, MetaLonDA, and LonGP

MetaLonDA (Metwally *et al.*, 2018) is proposed to identify the significant time intervals of differentially abundant microbial features in the longitudinal microbiome data. For a given microbial feature, MetaLonDA models the mapped read counts by a negative binomial (NB) distribution and fits the longitudinal profiles in each phenotypic group with NB smoothing splines. The microbial group difference per unit time interval is measured by the area ratio between two spline curves fitted in groups separately and the p-value for each time interval is calculated by a permutation test. LonGP (Cheng *et al.*, 2019) is an additive Gaussian process regression model designed for statistical analysis of longitudinal data and it incorporates multiple kernels to model time-varying random effects and non-stationary signals.

Note that MetaLonDA works on the time interval and provides the p-value for each time interval, rather than the overall study period. To obtain its overall significance for comparison, two strategies are considered to determine whether a taxon is significantly different or not. MetaLonDA_L: the taxon is determined as significantly different if any time interval $[t, t+1]$, $t = 1, \dots, T-1$, is significantly identified; MetaLonDA_G: the taxon is determined as significantly different if the time interval $[1, T]$ is significantly identified. All simulations related to the MetaLonDA method were analyzed with the R package MetaLonDA, in which we used the microbial counts, the NB smoothing spline, and 1000 permutations. For the LonGP, the taxon is claimed significantly different if the interaction between time point and group is selected using the additive Gaussian process. Note that LonGP provides the component relevance and the corresponding selection with a given threshold of the total relevance, not the testing p-value. Specifically, we employed the R package lgpr (Timonen *et al.*, 2021) to run the LonGP, in which we used the log-transformed count data and the threshold of 0.9.

We evaluated the proposed MTA method, Permuspliner, MetaLonDA, and LonGP

in terms of sensitivity and specificity in two simulation designs: (1) the same simulation design used in the main text; and (2) the simulation design based on the NB distribution used in the MetaLonDA method. In design 1, since the permutation procedure in MetaLonDA and the sampling procedure to determine the prior parameters in LonGP are both time-consuming, we simulated a small scale data with sample size $N = 20$ and time point $T = 10$ as an illustration without loss of generality. Figure S14 reports the estimated sensitivity and specificity of four methods in scenarios 1-3, respectively. As we discussed in the main Simulation results section, the proposed MTA has a superior and robust performance, while Permuspliner has the poorest performance in terms of sensitivity. For the MetaLonDA method, it is not surprising that neither MetaLonDA_L nor MetaLonDA_G has the comparable performance, because that MetaLonDA is based on the area under the microbial splines within a given time interval, similar to Permuspliner, where cross-over microbial trends cause the area between curves to balance out and reduce the power accordingly. LonGP does not have competitive sensitivities either, although it has close to 100% specificities.

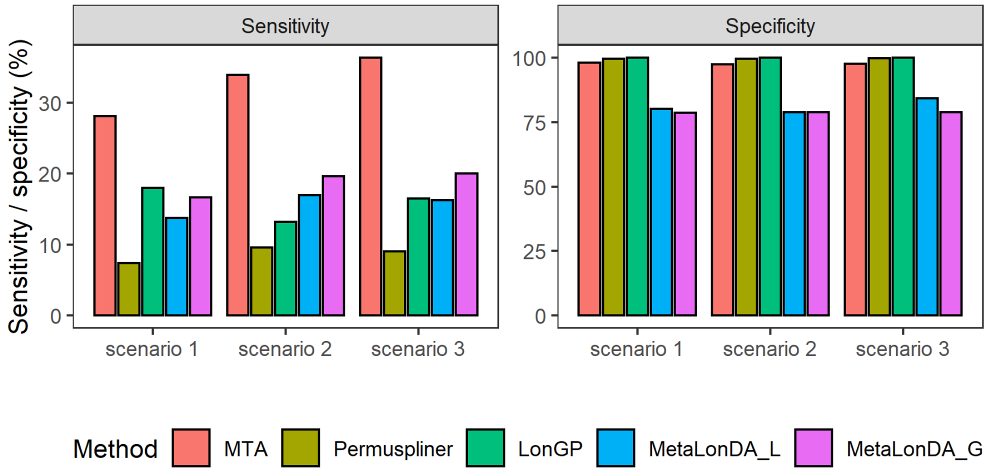

FIG S14 The estimated sensitivity and specificity for identifying the dominant taxa which contribute to the extracted trends with sample size $N = 20$ and the number of time points $T = 10$ in scenarios 1-3, respectively. Scenarios 1-3 were constructed as in the "Simulation design" section. MetaLonDA_L: the taxon is determined as significantly different if any time interval $[t, t+1]$, $t = 1, \ldots, T-1$, is significantly identified; MetaLonDA_G: the taxon is determined as significantly different if the time interval $[1, T]$ is significantly identified.

Second, we simulated data from the NB distribution using the parameters in the MetaLonDA method (Metwally *et al.*, 2018). Specifically, we considered the sample size $N = 20$ per group, the number of taxa $P = 20$, and the number of time points $T = 20$. Among 20 taxa, 4 were assumed significant in three time regions as in Metwally *et al.* (2018). Since the total depths varied across different samples, both the original counts and the rarefied counts were used for MetaLonDA and LonGP. The estimated sensitivity and specificity in Figure S15 show that the proposed MTA is able to identify all differentially abundant taxa successfully as well as has sufficient specificity. Permuspliner has commensurate performance. All LonGP and MetaLonDA methods suffer from low sensitivity.

In summary, the proposed MTA has robust and superior performance in the comprehensive simulation in terms of identifying the differentially abundant taxa, compared to all the competing methods including Permuspliner, MetaLonDA, and LonGP.

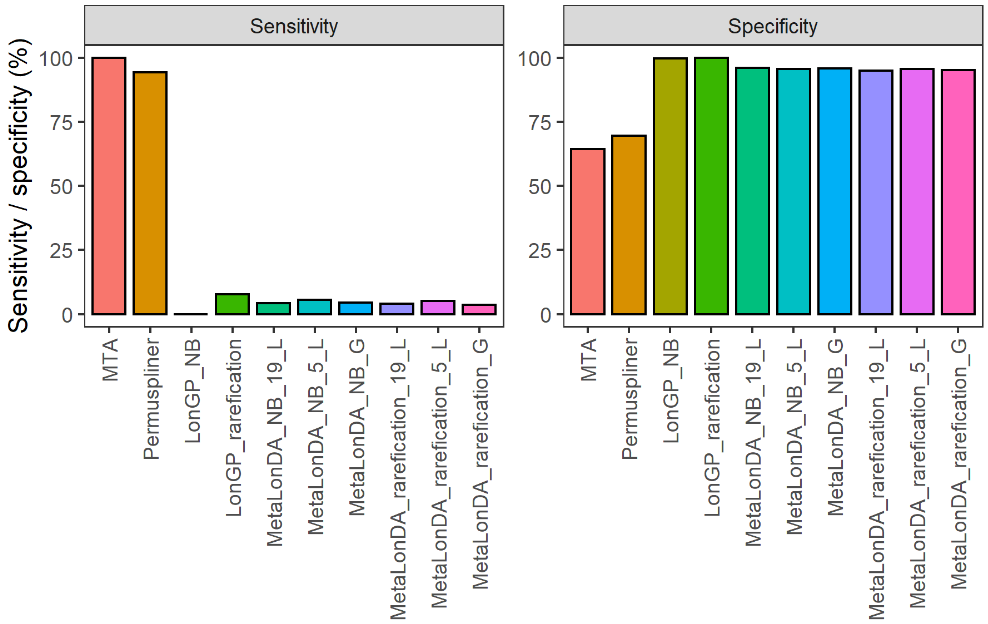

FIG S15 The estimated sensitivity and specificity for identifying the differentially abundant taxa with the sample size $N = 20$, the number of taxa $P = 20$ and the number of time points $T = 20$. The simulation data were generated from the NB distribution described in Metwally *et al.* (2018). LonGP_NB: LogGP employs the original counts generated from the NB distribution; LonGP_rarefication: LogGP employs the rarefied counts; MetaLonDA_NB_19_L: MetaLonDA employs the original counts and the taxon is determined as significantly different if any time interval $[t, t+1]$, $t = 1, \ldots, T$, is significantly identified; MetaLonDA_NB_5_L: MetaLonDA employs the original counts and the taxon is determined as significantly different if at least one of five predefined time intervals in Metwally *et al.* (2018) is significantly identified; MetaLonDA_NB_G: MetaLonDA employs the original counts and the taxon is determined as significantly different if the time interval $[1, T]$ is significantly identified; MetaLonDA_rarefication_19_L: MetaLonDA employs the rarefied counts and the taxon is determined as significantly different if any time interval $[t, t+1]$, $t = 1, \ldots, T$, is significantly identified; MetaLonDA_ rarefication_5_L: MetaLonDA employs the rarefied counts and the taxon is determined as significantly different if at least one of five predefined time intervals in Metwally *et al.* (2018) is significantly identified; MetaLonDA_ rarefication_G: MetaLonDA employs the rarefied counts and the taxon is determined as significantly different if the time interval $[1, T]$ is significantly identified.

Section S7: Sparsity evaluation of MTA

To evaluate whether and how the sparsity affects the performance of MTA, we modified the simulation design used in MetaLonDA and generated the longitudinal microbiome data from a zero-inflated NB distribution. Specifically, we simulated two types of zero inflation proportions. Type 1: the zero inflation proportion does not change over time, and is set at the values from 0.1 to 0.9 by 0.1, reflecting the sparsity from low to high. Type 2: the zero inflation proportion varies over time and was defined as $\text{proportion}_0 + I(t > 5) * 0.2$, $t = 1, \ldots, T$, where $\text{proportion}_0 = 0.1, 0.3$, or 0.5, and $I(\cdot)$ was the indicator function. For each type, two scenarios were considered as well. Scenario 1: all four causal taxa are sparse. Scenario 2: four randomly selected non-causal taxa are sparse. All the other simulation parameters are the same as those used in the main Simulation design section.

First, when the zero inflation proportion does not change (Type 1), the proposed MTA has power close to 1 with all zero inflation proportions in both scenarios. The estimated sensitivity and specificity are presented in Figure S16. We observe that the estimated sensitivity decreases, while the specificity increases as the zero inflation proportion increases in scenario 1. In scenario 2, both sensitivity and specificity are stable no matter what the sparsity is. In summary, the magnitude of sparsity in causal taxa affects the performance of the proposed MTA, but not the sparsity in non-causal taxa. In addition, MTA has lower sensitivities and higher specificities in scenario 1 than in scenario 2.

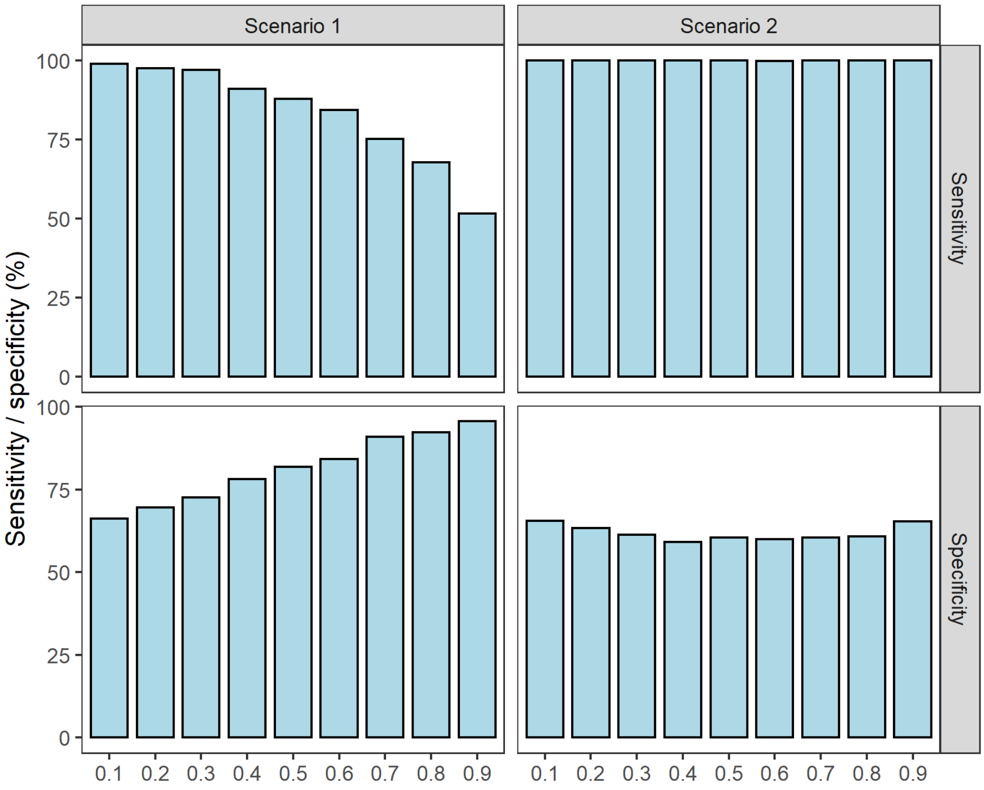

FIG S16 The estimated sensitivity and specificity for the MTA with various zero inflation proportions which are unchanged over time, in scenarios 1-2, respectively.

When the zero inflation proportion varies over time, the proposed MTA has power=1 with all zero inflation proportions in both scenarios. Similar to the pattern we observe in Type 1, Figure S17 shows that lower sensitivities and higher specificities in scenario 1 than in scenario 2 again. Unlike Type 1, the sensitivities are consistent with all proportion_0s, meanwhile, the specificity decreases as proportion_0s increases, in both scenarios.

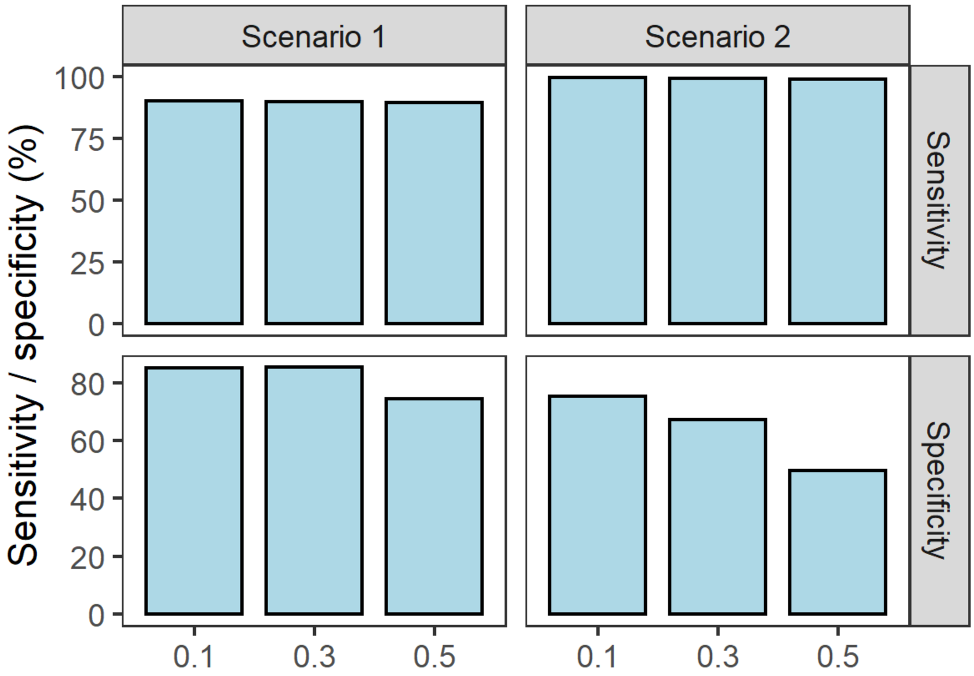

FIG S17 The estimated sensitivity and specificity for the MTA with various zero inflation proportions that vary over time, in scenarios 1-2 respectively.

References

Cheng, L., Ramchandran, S., Vatanen, T., Lietzén, N., Lahesmaa, R., Vehtari, A., and Lähdesmäki, H. (2019). An additive gaussian process regression model for interpretable non-parametric analysis of longitudinal data. *Nature communications*, **10**(1), 1–11.

Maxwell, O., Chukwudike, C. N., Chinedu, O. V., Valentine, C. O., and Paul, O. C. (2019). Comparison of different parametric methods in handling critical multicollinearity: Monte carlo simulation study. *Asian Journal of Probability and Statistics*, pages 1–16.

Metwally, A. A., Yang, J., Ascoli, C., Dai, Y., Finn, P. W., and Perkins, D. L. (2018).

Metalonda: a flexible r package for identifying time intervals of differentially abundant features in metagenomic longitudinal studies. *Microbiome*, **6**(1), 32.

Shields-Cutler, R. R., Al-Ghalith, G. A., Yassour, M., and Knights, D. (2018). Splinectomer enables group comparisons in longitudinal microbiome studies. *Frontiers in microbiology*, **9**, 785.

Timonen, J., Mannerström, H., Vehtari, A., and Lähdesmäki, H. (2021). lgpr: an interpretable non-parametric method for inferring covariate effects from longitudinal data. *Bioinformatics*. btab021.

Tvedebrink, T. (2009). dirmult: Estimation in dirichlet-multinomial distribution. *R Package Version 0.1*, **3**.

Zhang, Y. and Davis, R. (2013). Principal trend analysis for time-course data with applications in genomic medicine. *The Annals of Applied Statistics*, pages 2205–2228.

Zou, H., Hastie, T., and Tibshirani, R. (2006). Sparse principal component analysis. *Journal of computational and graphical statistics*, **15**(2), 265–286.
