# Supplementary material for: Microbial trend analysis for common dynamic trend, group comparison, and classification in longitudinal microbiome study
Source: BMC Genomics. 2021 Sep 15;22:667. doi: 10.1186/s12864-021-07948-w (PMC8442444; doi:10.1186/s12864-021-07948-w)
Supplement: Supplementary file 2 — Additional file 2FIG S1. The proposed MTA method for the comparison between case and control groups in scenario 1 with sample size N=30 and the number of time points T=10. (A) The microbial trend extracted by MTA represents the significant difference between case and control groups. (B) The average and standard error of the estimated factor scores for the dominant taxa that contribute to the extracted trend, respectively. FIG S2. The proposed MTA method for the comparison between case and control groups in scenario 2 with sample size N=30 and the number of time points T=10. (A) Two microbial trends extracted by MTA represent the significant difference between case and control groups. (B) The average and standard error of the estimated factor scores for the dominant taxa that contribute to those two trends, respectively. FIG S3. The proposed MTA method for the comparison between case and control groups in scenario 3 with sample size N=30 and the number of time points T=10. (A) The microbial trend extracted by MTA represents the significant difference between case and control groups. (B) The average and standard error of the estimated factor scores for the dominant taxa that contribute to the extracted trend, respectively. FIG S4. Empirical power for testing the difference between case and control groups with sample size N=20,30 and the number of time points T=10,20 under 1X and 2X magnitudes of perturbation, respectively. Here, (A) z=(0,0.3,0.45,0.2,−0.3,0.3,−0.3,− 0.2,−0.1,0)′ and z=(0,0.2,0.5,0.3,0.2,−0.2,−0.4,−0.4,−0.2,0.2,0.5,0.2,−0.3,−0.4,−0.2,0.2,0.4,0.2,0.1,0)′, (B) z=(0,0.1,0.2,0.3,0.4,0.3,0.2,0.1,0.1,0)′ and z=(0,0.05,0.1,0.2,0.2,0.2,0.3,0.3,0.2,0.2,0.2,0.2,0.3,0.3,0.2,0.2,0.2,0.2,0.1,0)′, for T=10, and 20, respectively. FIG S5. The estimated sensitivity and specificity for identifying dominant taxa which contribute to the extracted trends with sample size N=20,30 and the number of time points T=10,20 under 1X and 2X magnitudes of perturbation, respectively. [file 12864_2021_7948_MOESM2_ESM.pdf]

Additional file 2: Additional file 2 has 9 figures as below.

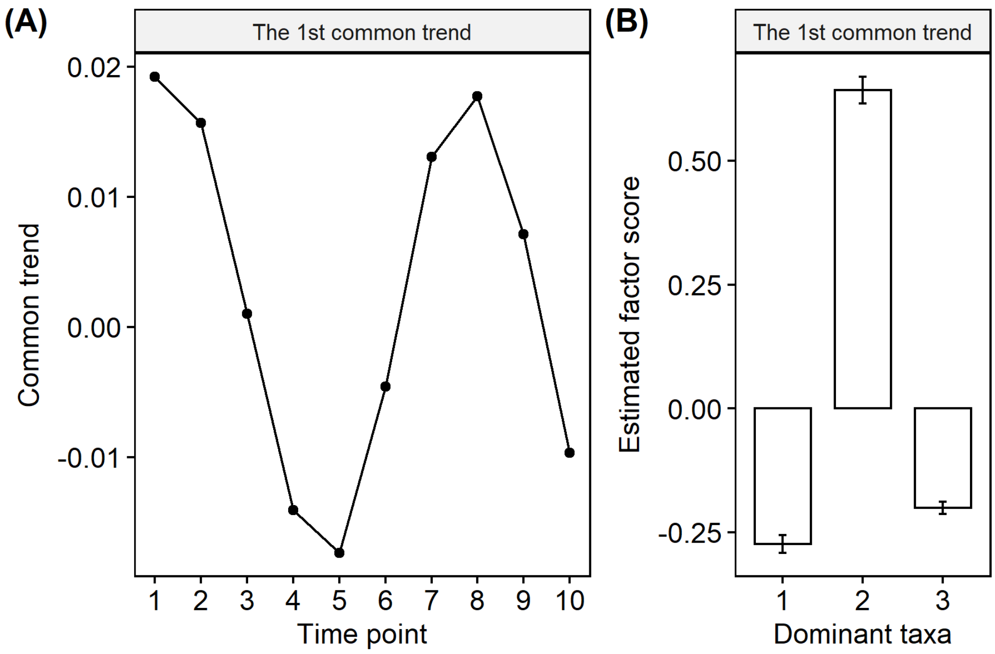

FIG S1 The proposed MTA method for the comparison between case and control groups in scenario 1 with sample size $N = 30$ and the number of time points $T = 10$. (A) The microbial trend extracted by MTA represents the significant difference between case and control groups. (B) The average and standard error of the estimated factor scores for the dominant taxa that contribute to the extracted trend, respectively.

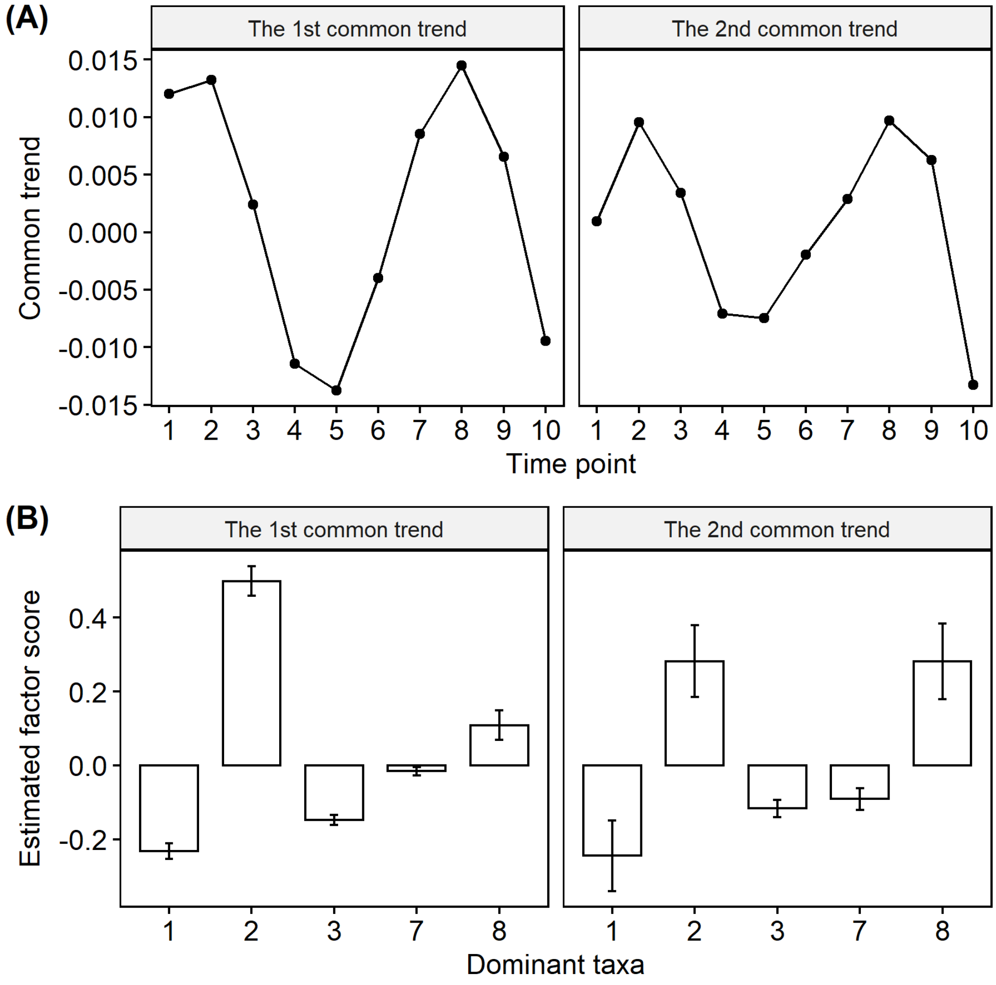

FIG S2 The proposed MTA method for the comparison between case and control groups in scenario 2 with sample size $N = 30$ and the number of time points $T = 10$. (A) Two microbial trends extracted by MTA represent the significant difference between case and control groups. (B) The average and standard error of the estimated factor scores for the dominant taxa that contribute to those two trends, respectively.

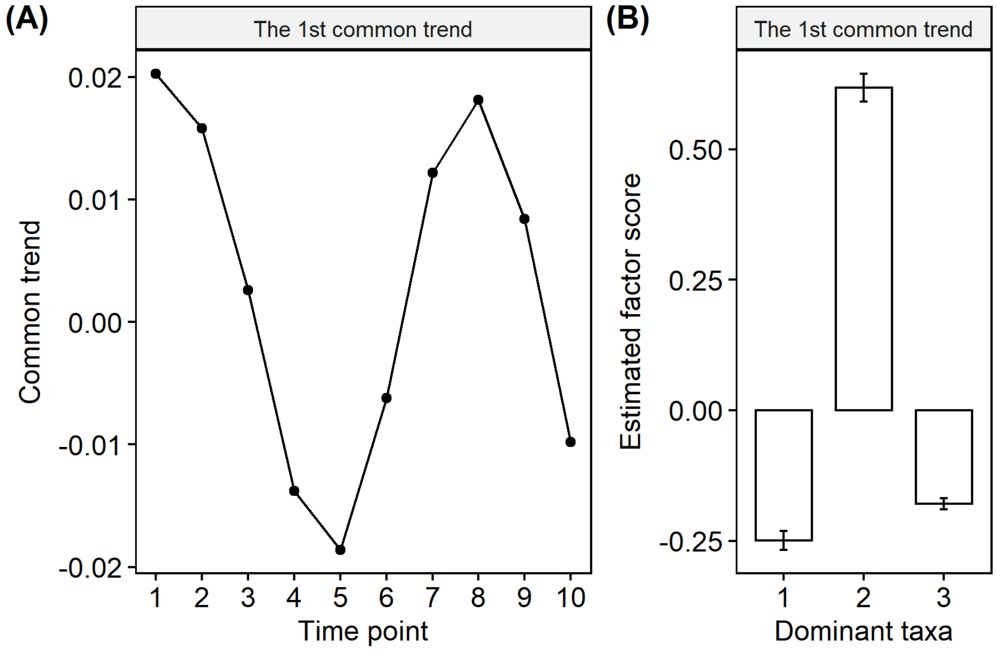

FIG S3 The proposed MTA method for the comparison between case and control groups in scenario 3 with sample size $N = 30$ and the number of time points $T = 10$. (A) The microbial trend extracted by MTA represents the significant difference between case and control groups. (B) The average and standard error of the estimated factor scores for the dominant taxa that contribute to the extracted trend, respectively.

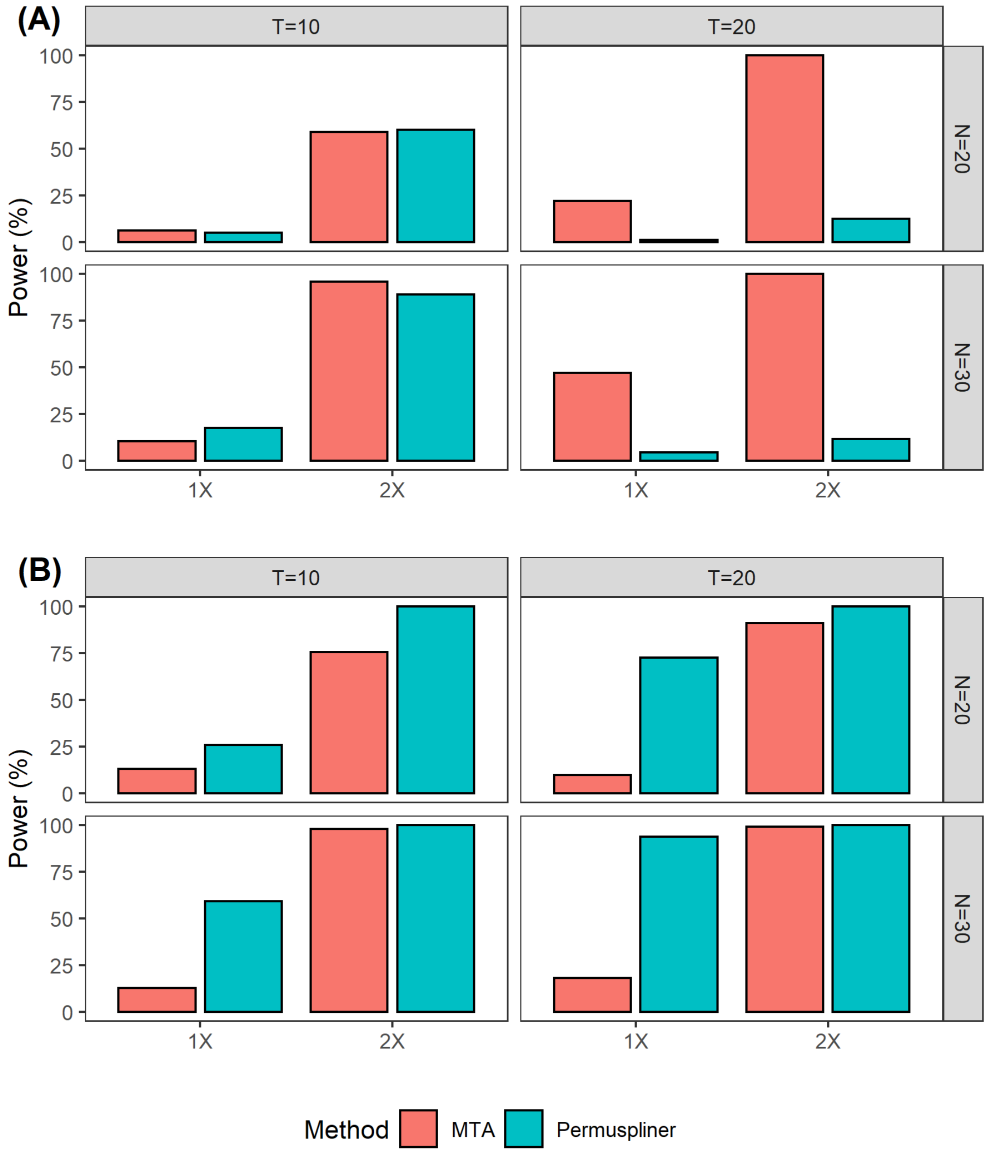

FIG S4 Empirical power for testing the difference between case and control groups with sample size $N = 20, 30$ and the number of time points $T = 10, 20$ under 1X and 2X magnitudes of perturbation, respectively. Here, (A) $\boldsymbol{z} = (0, 0.3, 0.45, 0.2, -0.3, 0.3, -0.3, -0.2, -0.1, 0)'$ and $\boldsymbol{z} = (0, 0.2, 0.5, 0.3, 0.2, -0.2, -0.4, -0.4, -0.2, 0.2, 0.5, 0.2, -0.3, -0.4, -0.2, 0.2, 0.4, 0.2, 0.1, 0)'$, (B) $\boldsymbol{z} = (0, 0.1, 0.2, 0.3, 0.4, 0.3, 0.2, 0.1, 0.1, 0)'$ and $\boldsymbol{z} = (0, 0.05, 0.1, 0.2, 0.2, 0.2, 0.3, 0.3, 0.2, 0.2, 0.2, 0.2, 0.3, 0.3, 0.2, 0.2, 0.2, 0.2, 0.1, 0)'$, for $T = 10$, and 20, respectively.

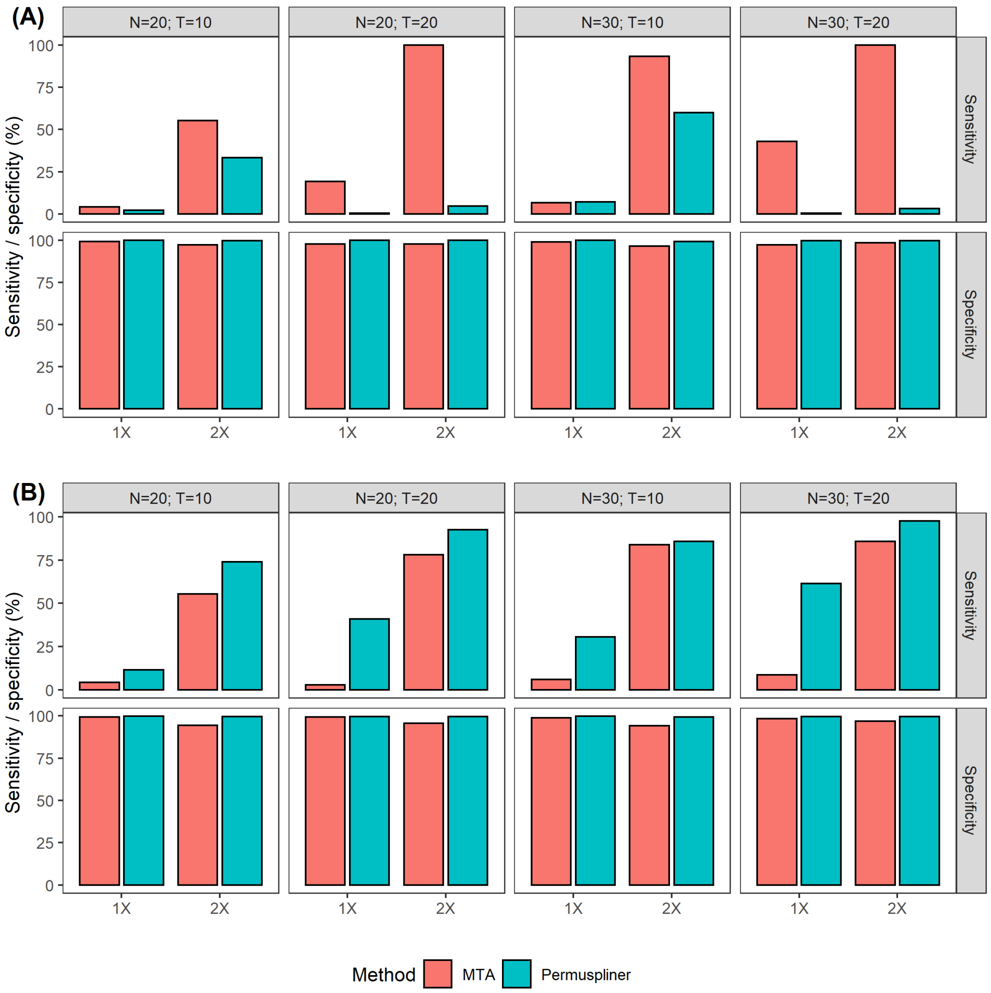

FIG S5 The estimated sensitivity and specificity for identifying dominant taxa which contribute to the extracted trends with sample size $N = 20, 30$ and the number of time points $T = 10, 20$ under 1X and 2X magnitudes of perturbation, respectively. Here, (A) $\boldsymbol{z} = (0, 0.3, 0.45, 0.2, -0.3, 0.3, -0.3, -0.2, -0.1, 0)'$ and $\boldsymbol{z} = (0, 0.2, 0.5, 0.3, 0.2, -0.2, -0.4, -0.4, -0.2, 0.2, 0.5, 0.2, -0.3, -0.4, -0.2, 0.2, 0.4, 0.2, 0.1, 0)'$, (B) $\boldsymbol{z} = (0, 0.1, 0.2, 0.3, 0.4, 0.3, 0.2, 0.1, 0.1, 0)'$ and $\boldsymbol{z} = (0, 0.05, 0.1, 0.2, 0.2, 0.2, 0.3, 0.3, 0.2, 0.2, 0.2, 0.2, 0.3, 0.3, 0.2, 0.2, 0.2, 0.2, 0.1, 0)'$, for $T = 10$, and 20, respectively.

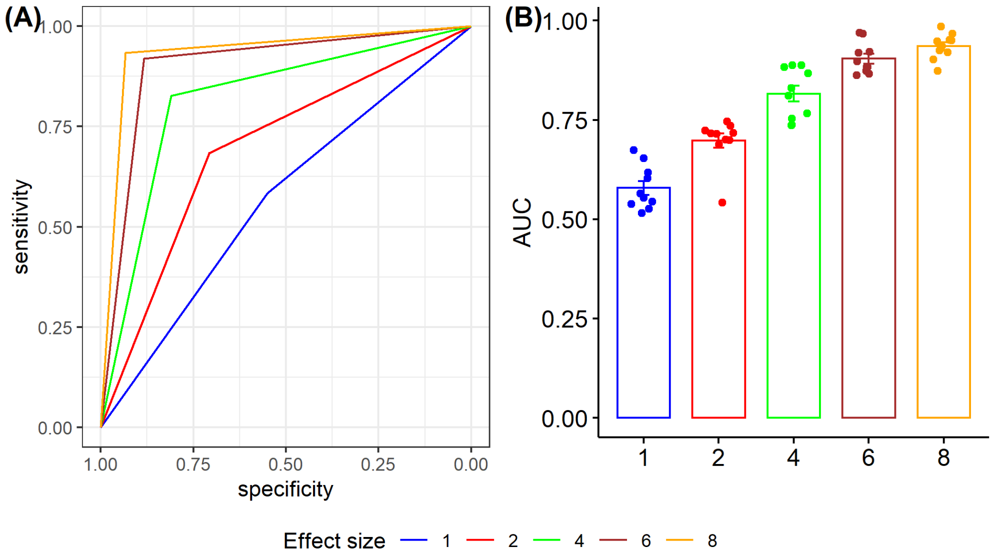

FIG S6 The classification performance of the proposed MTA framework with the number of time points $T = 10$. (A) The overall ROC curves. (B) The mean and standard error of AUCs under various effect sizes based on 10-fold CV.

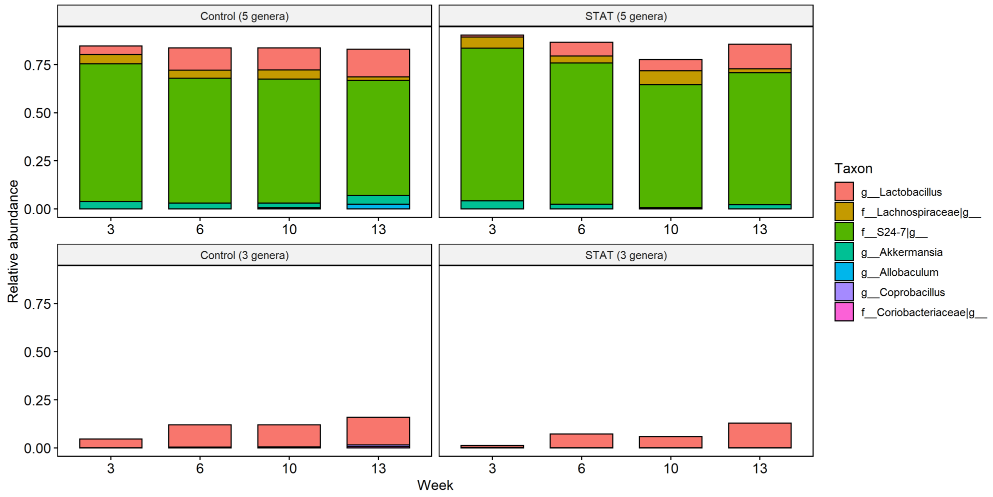

FIG S7 The relative abundances for the genera identified by MTA (5 genera) and Permuspliner (3 genera) at 3, 6, 10 and 13 weeks, respectively.

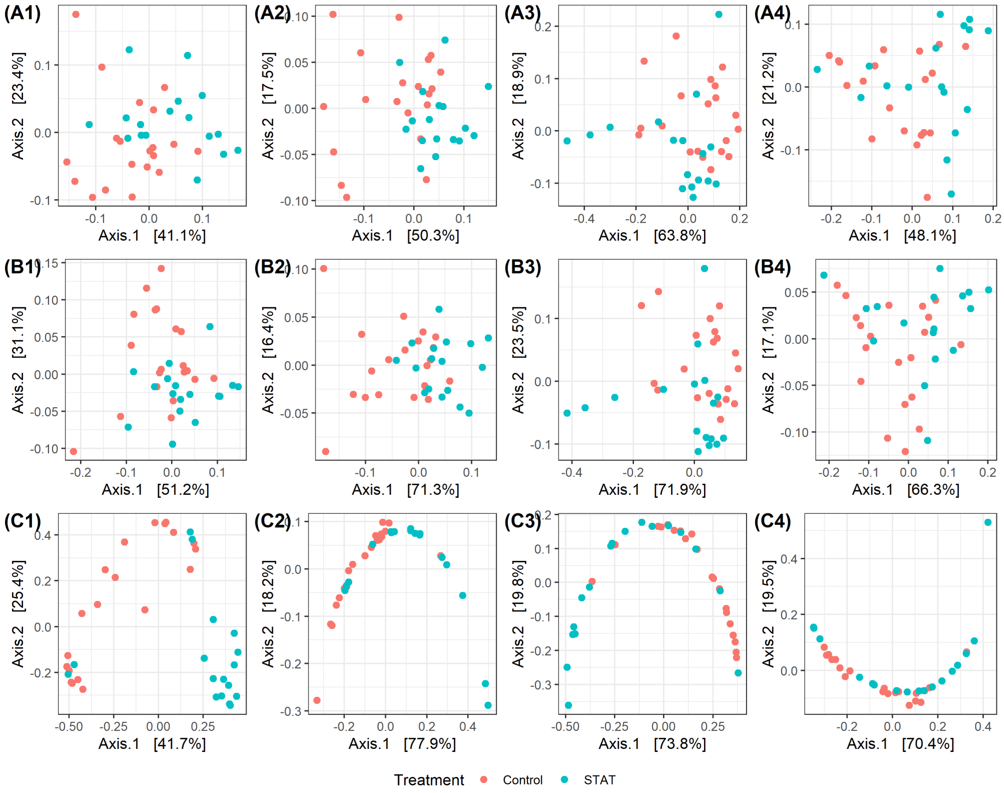

FIG S8 Beta diversity analysis for all male mice based on the Bray-Curtis dissimilarity index. The PCoA is evaluated based on: (A1)-(A4) 35 original genera; (B1)-(B4) 5 genera identified by the proposed MTA method; and (C1)-(C4) 3 genera identified by the competing method Permuspliner at 3, 6, 10 and 13 weeks, respectively. Points represent samples.

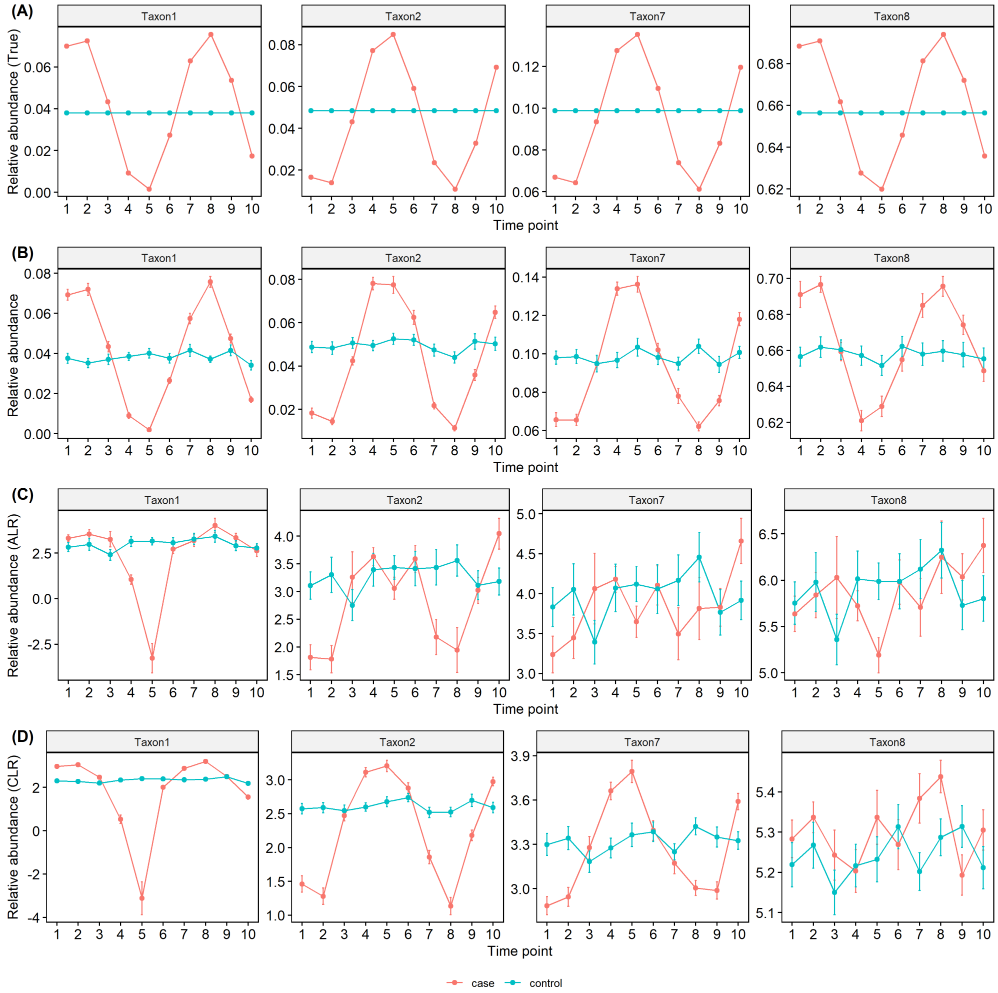

FIG S9 Evaluation of the transformations affect on the longitudinal trends of the causal taxa that contribute to group difference in scenario 1 with sample size $N = 30$ and the number of time points $T = 10$ respectively. (A) The true relative abundance time series of the causal taxa with equation (1). (B) The mean and standard error of the relative abundances without none transformation. (C) The mean and standard error of the relative abundances after ALR (additive log-ratio transformation), with the last one taxon being the reference. (D) The mean and standard error of the relative abundances after CLR (centered log-ratio transformation).
